# Supplementation with a Polyphenol-Rich Extract, PerfLoad^®^, Improves Physical Performance during High-Intensity Exercise: A Randomized, Double Blind, Crossover Trial

**DOI:** 10.3390/nu9040421

**Published:** 2017-04-24

**Authors:** Julien Cases, Cindy Romain, Cristian Marín-Pagán, Linda H. Chung, José Miguel Rubio-Pérez, Caroline Laurent, Sylvie Gaillet, Emmanuelle Prost-Camus, Michel Prost, Pedro E. Alcaraz

**Affiliations:** 1 Fytexia, Innovation and Scientific Affairs, 34350 Vendres, France; cromain@fytexia.com; 2 Research Center in High Performance Sport, UCAM Universidad Católica de Murcia, 30107 Murcia, Spain; cmarin@ucam.edu (C.M.-P.); lhchung@ucam.edu (L.H.C.); jmrubio@ucam.edu (J.M.R.-P.); palcaraz@ucam.edu (P.E.A.); 3 UMR 204 Nutripass, Institut de Recherche pour le Développement, Université de Montpellier, 34095 Montpellier, France; caroline.laurent@univ-montp2.fr (C.L.); sylvie.gaillet@univ-montp2.fr (S.G.); 4 Laboratoires Spiral, 21560 Couternon, France; camusprost@hotmail.fr (E.P.-C.); michelprost.spiral@wanadoo.fr (M.P.)

**Keywords:** preworkout, anaerobic power, pulse pressure, oxidative stress, Wingate test

## Abstract

Workout capacity is energy-production driven. To produce peak metabolic power outputs, the organism predominantly relies more on anaerobic metabolism, but this undoubtedly has a negative and limiting impact on muscle function and performance. The aim of the study was to evaluate if an innovative polyphenol-based food supplement, PerfLoad^®^, was able to improve metabolic homeostasis and physical performance during high-intensity exercises under anaerobic conditions. The effect of a supplementation has been investigated on fifteen recreationally-active male athletes during a randomized, double-blind and crossover clinical investigation. The Wingate test, an inducer of an unbalanced metabolism associated to oxidative stress, was used to assess maximum anaerobic power during a high-intensity exercise on a cycle ergometer. Supplementation with PerfLoad^®^ correlated with a significant increase in total power output (5%), maximal peak power output (3.7%), and average power developed (5%), without inducing more fatigue or greater heart rate. Instead, oxidative homeostasis was stabilized in supplemented subjects. Such results demonstrated that PerfLoad^®^ is a natural and efficient solution capable of, similarly to training benefits, helping athletes to improve their physical performance, while balancing their metabolism and reducing exercise-induced oxidative stress.

## 1. Introduction

Workout capacity is determined by the ability to synchronise interactions between the cardiovascular, respiratory, skeletal-muscular, and nervous systems [1]. Additionally, the characteristics of the exercise predominantly dictate the preferred metabolic pathway used to yield sufficient energy and, consequently, affect overall performance. Energy production is both time- and intensity-related, depending on the energy system involved: phosphagenic (high intensity and short duration), glycolytic (moderate intensity and short duration), or oxidative (low intensity and long duration) [2,3]. Accordingly, when high peak metabolic power outputs are to be achieved, the organism predominately relies more on anaerobic metabolism; as an adaptive response, the exerting muscles prioritise two pathways of stored energy, both accessible without oxygen: the phosphagen system and anaerobic glycolysis. Such anaerobic functioning produces energy more rapidly, but this is at the cost of increased accumulation of unfavourable end-products, mainly lactate and reactive oxygen species, which may have a negative and limiting impact on muscle function and performance [4]. High-intensity training is commonly used to help reduce the production of fatigue-associated metabolites, increase better metabolic buffering [5,6,7], as well as lower the neuromuscular fatigue [8]. In addition, it improves both aerobic and anaerobic systems [9]. Working at high-intensity levels also challenges the organism’s capability to minimize oxidative stress [10].

Pre-workout supplementation has become a significant nutritional approach in recent years. Several bioactive components [11,12,13] may have a positive effect on metabolic activity, which benefits strength and power and overall performance during physical exercise. PerfLoad^®^ is an innovative food supplement, composed of polyphenols, which has been designed to improve metabolic homeostasis during high-intensity exercises. Although some studies have examined the effect of polyphenols on blood flow [14,15,16,17], it is not known how polyphenols may affect physical performance, cardiovascular, and metabolic responses during high-intensity activities. 

Therefore, the aim of the present clinical study was to investigate whether supplementation with PerfLoad^®^ could improve physical performance during high-intensity physical exercise in anaerobic conditions while sustaining physiological and biochemical processes of recreationally-active athletes.

## 2. Materials and Methods 

### 2.1. Ethics Statement

The protocol of the study was approved by the Ethics Committee at the Catholic University of Murcia on 30 October 2015, protocol number CFE-PERFLOAD-58/15, and conducted according to the guidelines laid down in the Declaration of Helsinki [18] and in compliance with good clinical practices defined in ICH Harmonized Tripartite Guidelines [19]. All participants were informed about the operating procedures of the clinical trial and they agreed to sign a written informed consent form before entering the study.

### 2.2. Study Population

Twenty healthy recreationally-active (i.e., not performing more than three days of moderate-intensity activity per week) male volunteers in comparable physical condition, a minimum of 20 years old, were recruited through advertisements in Murcia, Spain. Any individuals usually involved in high-intensity training or with a history of cardiorespiratory problems or chronic illness were not enrolled. In addition, individuals with a history of orthopaedic injury or surgery within the past year or any physical condition considered a contraindication to the type of exercise performed in the study were excluded. Additionally, those affected by any medical condition or using any medication, nutritional product, dietary supplement, or program which might interfere with the conduct of the study or place the subject at risk, were not included. Furthermore, individuals could not participate if they had any food allergy to grape, green tea, pomegranate, or corn.

### 2.3. Test Supplement

PerfLoad^®^, supplied by FYTEXIA (France), is principally obtained by alcohol and water extraction of grape (*Vitis vinifera* L.) and pomegranate (*Punica granatum* L.), and by water extraction of green tea (*Camellia sinensis* L. Kuntze). PerfLoad^®^ provides bioactive compounds, especially polyphenols, from flavonoid, ellagitannin, and stilbenoid families, and natural components of the methylxanthine family. PerfLoad^®^ complies with regulations on food contaminants and on banned and prohibited substances. The placebo product was 100% maltodextrin, which is polyphenol- and methylxanthine-free. Both PerfLoad^®^ and the placebo were supplied in 500-mg capsules of identical appearance and flavour.

The supplement was analysed by means of high-peformance liquid chromatography (HPLC). An Agilent HPLC 1260 apparatus (Singapore) (Openlab CDS chemstation edition software (Santa Clara, CA, USA) was used) coupled with a diode array detector was used. Separations were carried out by means of a Zorbax Stablebond SB-C18 column (4.6 × 2 mm; 5 µm particle size; New Port, DE, USA). To detect different phenolic classes, two different analytical methods were adopted: one for bioflavonoids and trans-resveratrol and one for punicalagins. Data analyses were performed as described below.

For bioflavnonoids and trans-resveratrol, mobile phase A consisted of 6% acetic acid, mobile phase B was 5% acetic acid and 30% acetonitrile, and mobile phase C was 100% acetonitrile. The program was as follows: (a) 5 min 100% A; (b) 5 to 10 min linear gradient from 0% to 40% B; (c) 10 to 15 min linear gradient from 40% to 60% B; (d) 15 to 20 min linear gradient from 60% to 75% B; (e) 20 to 25 min linear gradient from 75% to 90% B; (f) 25 to 30 min linear gradient from 90% to 100% B; (g) 30 to 35 min linear gradient from 0% to 100% C; (h) 35 to 40 min 100% C; (i) 40 to 45 min linear gradient from 0% to 100% A. Simultaneous monitoring was performed at 280 and 310 nm at a flow rate of 1 mL/min and an injection volume of 25 µL.

For punicalagins, the mobile phase consisted of 0.1% orthophosphoric acid and mobile phase C was 100% methanol. The program was as follows: (a) 0 to 15 min linear gradient from 5% to 10% B; (b) 15 to 30 min linear gradient from 10% to 30% B. Monitoring was performed at 260 nm at a flow rate of 0.8 mL/min and an injection volume of 5 µL.

Bioflavonoids, punicalagins, trans-resveratrol, and caffeine were respectively determined as catechin at 280 nm, punicalagins at 260 nm, trans-resveratrol at 310 nm, and caffeine at 280 nm.

### 2.4. Study Design

The study was a randomized, double-blind, cross-over and placebo-controlled pilot clinical trial. Seven days prior to the supplementation period, volunteers underwent familiarization with the exercise protocol. Following inclusion, they were randomly assigned, using a random allocation sequence generated by a computerized random number generator, to one of two groups. At day one of the investigation, subjects received their supplementation and were instructed to ingest, acutely, 1000 mg of the supplement in two 500-mg capsules of either PerfLoad^®^ or placebo, in the presence of the investigator 60 min before the start of the exercise protocol. Simultaneously, they received a standardized and polyphenol- and antioxidant-free breakfast consisting of a ham and cheese sandwich, six “Maria” cookies, and one glass of water. After a five-week wash-out period, each volunteer was switched to the other supplementation group for a second investigation period. On days without exercise, volunteers were instructed to continue practicing their usual physical recreational activities. 

### 2.5. Exercise Protocol

The exercise protocol consisted of 4 × 30-s bouts of cycling (Wingate test; WAnT) at all-out intensity, which was performed on a cycle ergometer (Monark Ergomedic 894E Peak Bike, Vansbro, Sweden). For every bout, the breaking force was constant and individualized for each volunteer, corresponding to 7.5% of the body mass. Prior to the first repetition, a 5-min standard warm-up protocol was performed followed by a 1-min passive recovery period [20]. The rest period between the first and second sprints and between the third and fourth was 4 min long. Between the second and third sprints, the recovery period lasted for 5 min.

### 2.6. Primary Outcome

The primary outcome was the anaerobic mechanical power production performed at an “all-out” pace (i.e., maximal) during the first 30-s sprint. Anaerobic peak power (PP: highest anaerobic mechanical power generated within 30-s bout), total anaerobic work capacity (TAWC), anaerobic average power (AP), and anaerobic fatigue index (FI: percentage of decline in power) were also determined for the first bout. The second, third, and fourth sprints were performed with the aim of inducing greater oxidative stress [21,22].

### 2.7. Secondary Outcome

On the testing days, secondary outcomes were monitored at different points. In the pre-test period, both blood pulse pressure and heparinized blood samples were measured and analysed 60 min before the volunteers performed the exercise protocol. These procedures were then repeated (i) just before the start of the first sprint; (ii) following the completion of the fourth bout; and (iii) again in the recovery period, 60 min after subjects completed the last sprint. Heart rate was taken 30 s after the end of the Wingate test. The average heart rate from the post-30 s–60 s time interval was calculated.

Similarly, both total circulating antioxidant defences and total circulating antioxidant reserves were monitored in fresh heparinized blood using the KRL (Kit Radicaux Libres) [23,24,25,26,27,28] and RESEDA (RESErves Défenses Antioxydantes) tests (Spiral Laboratories, 21560 Couternon, France). The KRL test [29,30] allows the dynamic evaluation of the overall antioxidant defense potential of an individual. The RESEDA test [31] is based on the same principle with a step of releasing biologically active and potentially antioxidant molecules in reserve as: R1 (glucosides), R2 (sulphates), and R3 (glucuronides).

Similarly, lactate dehydrogenase (LDH) was measured using a colorimetric commercial kit (ab102526, Abcam, Cambridge, MA, USA) to both confirm exercise-induced haemolysis of red blood cells and to evaluate supplement protection. Finally, frozen (stored at −80 °C) blood samples were analysed for specific antioxidant enzymes. Activities of superoxide dismutase (SOD) and glutathione peroxidase (GSH-PX) were performed in plasma samples using colorimetric commercial kits (respectively, ab65354 and ab102530 kits, Abcam, Cambridge, MA, USA), and the activity of catalase was performed in haemolysed erythrocytes using a colorimetric commercial kit (ab83464, Abcam, Cambridge, MA, USA).

### 2.8. Statistical Analysis

Datasets were analysed using Statview software version 4.51.1 (Abacus Concepts, Berkeley, CA, USA). The data are expressed as mean ± standard deviation (SD). Changes within and between groups during the length of the studied period of the crossover protocol were analyzed using a paired Student’s *t*-test. Statistical significance was set at *p* < 0.05.

## 3. Results

### 3.1. Characterisation of the Phenolic Profile of the Supplement

**Figure 1:**
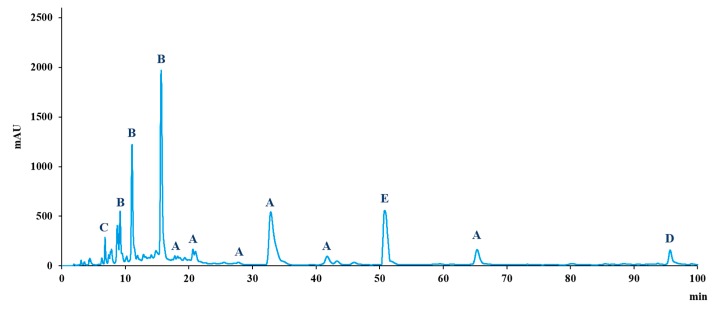
HPLC polyphenolic profile of the supplement at 260, 280, and 310 nm (A = bioflavonoids; B = punicalagins; C = gallic acid; D = trans-resveratrol; E = caffeine).

The total bioactive content was measured to correspond to 41 g/100 g dry matter. Bioflavonoid content was measured at 17 g/100 g, punicalagin content was measured at 10 g/100 g, caffeine content was measured at 12 g/100 g, and trans-resveratrol content was measured at 2 g/100 g (Figure 1). For a 1000 mg dose, it corresponds to 410 mg of ingested bioactives, i.e., 290 mg of various polyphenolic bioactives and 120 mg of caffeine (Table 1). 

### 3.2. Participant Characteristics

**Figure 2:**
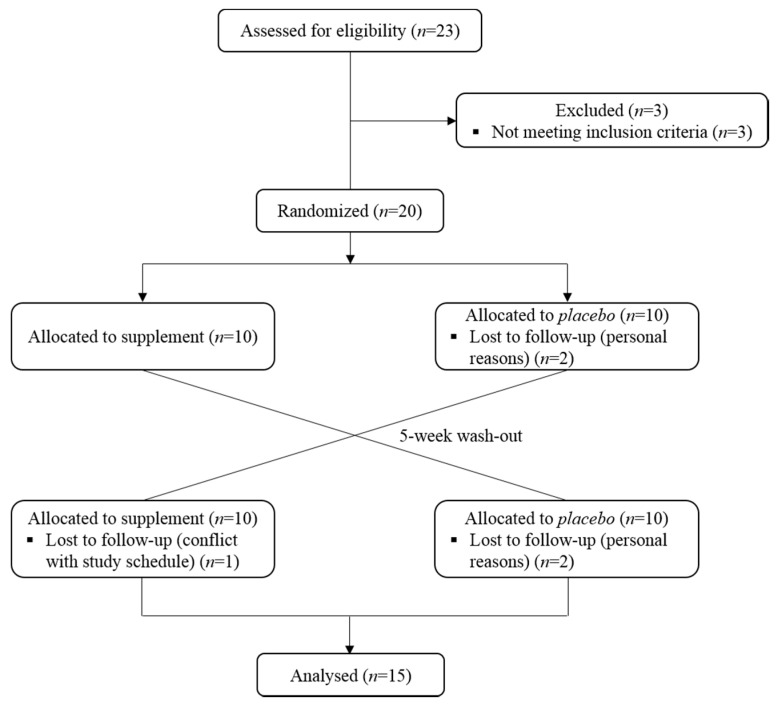
CONSORT (Consolidated Standards of Reporting Trials) flowchart of participants during the study intervention.

Twenty-three volunteers were assessed for eligibility and three of them did not meet the inclusion criteria and were excluded from participation in the study. Among the twenty volunteers recruited in the study, four did not report to the Research Center for personal reasons and one did not complete the exercise protocol due to conflict with their study schedule (Figure 2).

Fifteen participants completed the entire protocol and were included in the analysis. Baseline anthropometric characteristics of volunteers are summarized in Table 2.

### 3.3. Power Production

**Figure 3:**
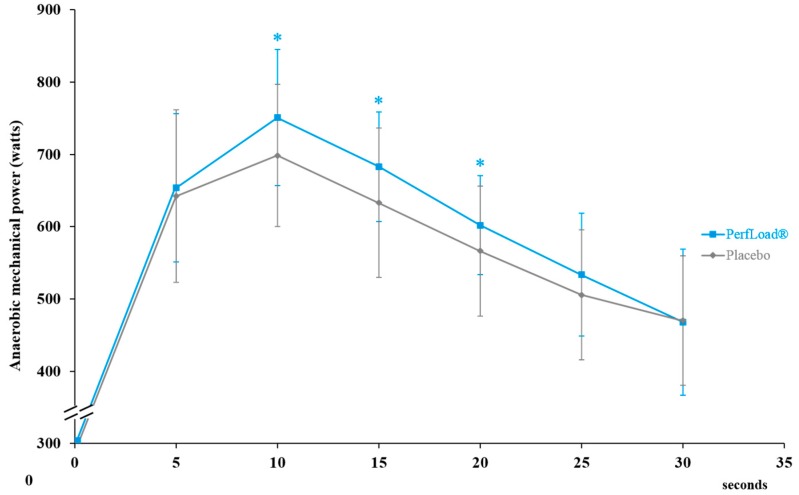
Anaerobic mechanical power (W) developed during a 30-s Wingate bout and representing the total anaerobic work capacity (J/30 s). Values are means ± SD, *n* = 15 (placebo) or *n* = 15 (PerfLoad^®^). * Superscript indicates an intergroup difference between PerfLoad^®^ and placebo, *p* < 0.05.

TAWC significantly increased by 5.0% in the supplemented group compared to the placebo population (*p* = 0.025), representing a production of an additional 876 Joules in 30 s group (Figure 3).

Similarly, PP of the supplemented subjects significantly improved by 3.7% (*p* = 0.048), and the production of AP was significantly enhanced by 5.0% (*p* = 0.025), compared to those who received the placebo (Table 3). In both groups, no significant difference was demonstrated in the anaerobic fatigue index, FI, with respective values of 39% and 38% (*p* = 0.395) for the supplement and placebo groups (Table 3).

Similarly, heart rate, which was measured just after the end of the exercise of power production, revealed no significant between-group difference, with 132 and 129 beats per minute (*p* = 0.344) (Table 3).

### 3.4. Blood Pulse Pressure

**Figure 4:**
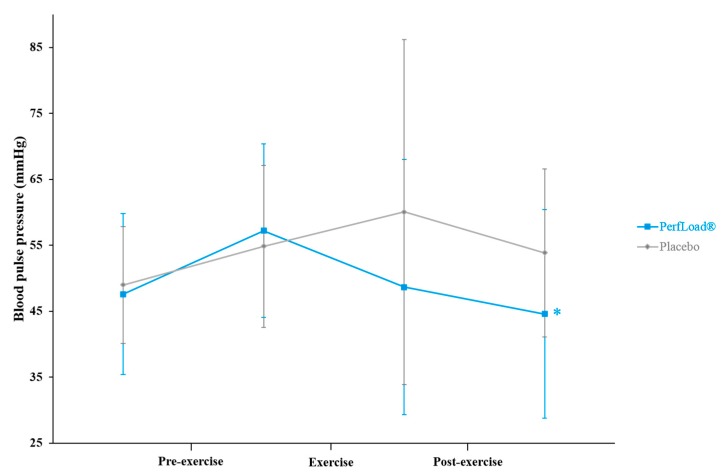
Blood pulse pressure (mmHg) measured 60 min before and just before (Pre-exercise), just before and just after (Exercise), just after and 60 min after (Post-exercise) a 30-s Wingate bout. Values are means ± SD, *n* = 15 (placebo) or *n* = 15 (PerfLoad^®^). * Superscript indicates an intergroup difference between PerfLoad^®^ and placebo, *p* < 0.05.

Both groups demonstrated a slight increase in blood pulse pressure (BPP) just before the start of the first sprint (Figure 4). While the placebo group experienced an additional elevation of BPP at the end of the exercise, supplemented subjects exhibited a decrease, reaching the basal level they had 60 min before; at 11.4 mmHg (*p* = 0.095), the difference between both groups represents a significant trend. Sixty minutes after the end of the exercise, the difference between both groups was significant at 9.3 mmHg (*p* = 0.034).

### 3.5. Protection of Red Blood Cells

**Figure 5:**
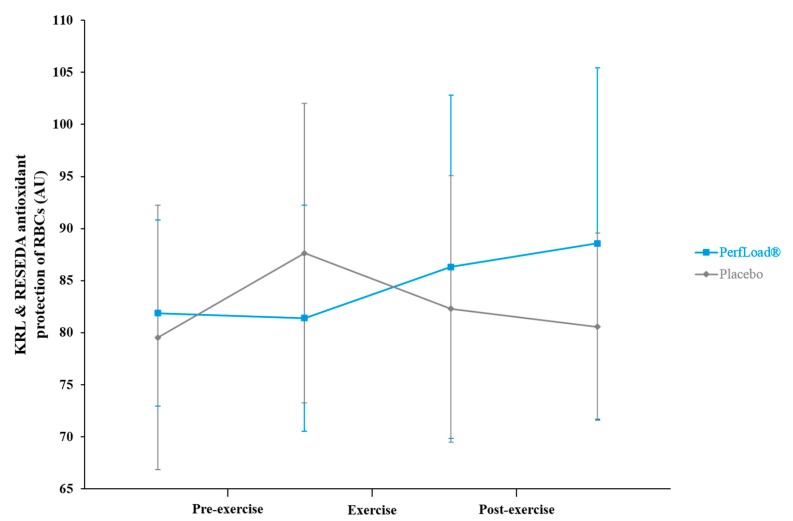
KRL and RESEDA global antioxidant protection of red blood cells (RBCs) (arbitrary units) measured 60 min before and just before (Pre-exercise), just before and just after (Exercise), and just after and 60 min after (Post-exercise) a 30-s Wingate bout. Values are means ± SD, *n* = 15 (placebo) or *n* = 15 (PerfLoad^®^). * Superscript indicates an intergroup difference between PerfLoad^®^ and placebo, *p* < 0.05.

The KRL and RESEDA test assays revealed that while the KRL level for subjects in the placebo group significantly increased by 10.2% (*p* = 0.021) during the 60-min period of pre-exercise, a respective decrease of 0.6% (*p* = 0.437) within the supplemented population for the same period demonstrated the absence of any significant change in the KRL level for the group (Figure 5).

Applying the test to blood samples taken immediately after the exercise period, a significant 6.1% (*p* = 0.040) decrease in KRL level was found within the placebo group, whereas an opposite trend seemed to be experienced by the volunteers who received the supplement with a 6% (*p* = 0.121) increase in this marker of protection. Finally, during the recovery period, the KRL protection values for individuals who received the placebo continued to decline by 2.1% and nearly reached their basal level, while the values in those supplemented with PerfLoad^®^ continued to enrich by 2.6% to reach a strong increasing trend of 8.2% compared to their basal level (*p* = 0.084).

### 3.6. Antioxidant Enzymes and LDH

**Figure 6:**
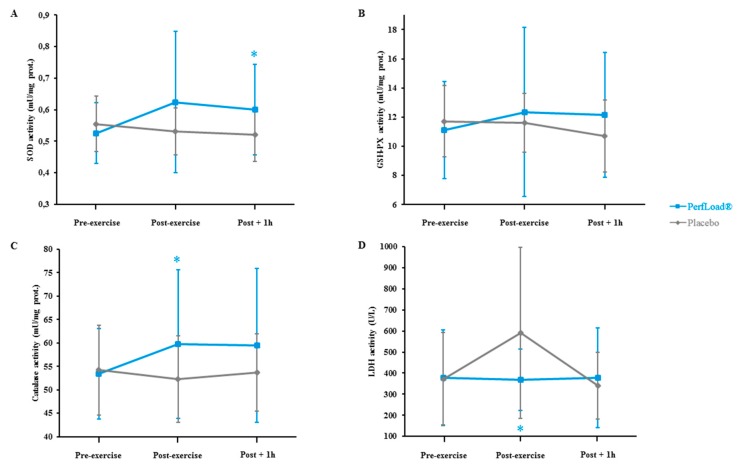
SOD (**A**), GSH-PX (**B**), Cat (**C**), and LDH (**D**) activities measured just before (Pre-exercise), just after (Exercise), and 60 min after (Post-exercise) a 30-s Wingate bout. Values are means ± SD, *n* = 15 (placebo) or *n* = 15 (PerfLoad^®^). * Superscript indicates an intergroup difference between PerfLoad^®^ and placebo, *p* < 0.05.

During the exercise period, SOD activity remained stable within the placebo group, but started to rapidly weaken by 4.3% during the recovery period (Figure 6A). In parallel tests, immediately after the first sprint, the value of plasmatic SOD in subjects supplemented increased by 18.7% and stabilized at 14.1% at the end of the recovery period. With a value of 20.3%, the difference in SOD activity between the groups after 60 min of recovery was significant (*p* = 0.043).

Similarly, with a decrease of 0.9%, GSH-PX activity measured immediately after the exercise period within the placebo group demonstrated no change, but started to decrease during the recovery phase (Figure 6B). At the same time, GSH-PX activity in supplemented volunteers increased by 11.1% just after the resistance workload, and remained stable until the end of the recovery period. The difference in GSH-PX activity reached among both groups was established at 17.9% (*p* = 0.106).

With respect to catalase, the scenario was quite similar. Within the placebo population, the enzyme activity slightly decreased by 3.6% consecutively with the power production exercise, and stabilized thereafter (Figure 6C). Among supplemented subjects, the enzyme increased by 11.9% after the physical exercise and stabilized until the end of the recovery period. Thus, at the end of the exercise period, the difference of variation between both groups reached 15.5% (*p* = 0.047), which almost stabilized until the recovery period with a strong trend of a difference of 12.4% (*p* = 0.085).

Finally, regarding LDH released in the plasma, a significant increase of 59.2% (*p* = 0.016) was found in the placebo-supplemented group just at the end of the exercise protocol, while no modification in LDH levels during the same period was evident in supplemented subjects (Figure 6D). At the end of the recovery period, LDH of the placebo group returned to basal levels. 

## 4. Discussion

There are several physiological factors that limit performance during a 30-s WAnT. Extremely intense sprint exercise cannot be sustained for longer than 60 s due to the bioenergetics of all-out efforts [32]. In the course of our investigation, we demonstrated that volunteers supplemented with PerfLoad^®^ were able to increase their TAWC by 5.0%, corresponding to an overproduction of 876 Joules in 30 s. Their PP production improved by 27 Watts, which represents a significant increase in power output. Finally, the AP produced was enhanced by 29 Watts/s, demonstrating that the significant improvement of power production is sustainable during the whole time of the exercise. To reach comparable performances, athletes usually have to undergo very specific physical training and/or be supplemented with synthetic aids [12,13]. This was confirmed in cycling athletes who were trained for a six-week period to gain 600 Joules of production [33]. In another study, volunteers subjected to a 30-s high-intensity exercise and supplemented with creatine phosphate, experienced only a slight increase in workload of 3.2% compared to the placebo group [34]. Thus, PerfLoad^®^ supplementation is apparently able to rapidly and significantly increase performance during an intense anaerobic effort. Since such results have not been previously described by the support provided by one of its single ingredients alone at a similar dose, the benefits of supplementation are possibly associated to synergistic formulation. In the example of caffeine, despite the fact that it is often referred to as a nutritional ergogenic aid, many researchers have demonstrated that the methylxanthine compound, at low to moderate levels (<5 mg/kg BW), fails to improve performance during WAnT [35,36,37].

Another particularly interesting endpoint observed, despite the higher power outputs developed with the supplement, is that fatigability and heart workload were, nevertheless, not affected more than with the placebo. Regarding the latter, it was even demonstrated that the supplementation could provide greater protection to the heart from increased workload. Indeed, despite a similar increase in heart rate during intense exercise-induced stress in both groups, when one considers the blood pulse pressure, a strong biomarker for heart workload [38,39,40], after supplementation the circulatory system required less strength (−11.4 mmHg), which probably spared tissues more from heart shear stress. Moreover, this was sustainable during the 60-min recovery period with a −9.3 mmHg difference compared to the placebo group. One possible explanation is a significant decrease in vessel resistance within the supplemented group. Indeed, several authors demonstrated the capacity of polyphenolic compounds for improving endothelial function [41]. Notably, green tea, grape, and pomegranate consumption, whether short- or long-term, has been associated with improved endothelial function in clinical trials [42,43,44]. The proposed main mechanisms by which polyphenols would mediate improved flow-mediated dilation, though this was not measured in the current study, are their ability to modulate the bioavailability of nitric oxide (NO) in the endothelium through activation of endothelial nitric oxide synthase (eNOS), and the inhibition of nicotinamide adenine dinucleotide phosphate (NADPH) oxidase, together leading to an endothelium-dependent relaxation [45].

During exercise and muscle contraction, antioxidant stores may become depleted, leaving the body susceptible to oxidative damages [46,47]. This was confirmed with results from KRL and RESEDA tests within the placebo population. Despite having mobilized their antioxidant defence mechanisms during the pre-exercise period, in the span of only 30 s, the high-intensity effort consumed all of the freed potential, and consequently decreased as many antioxidants from the reserves [24]. As opposed to the placebo group, subjects supplemented showed no need to mobilize more defences before the start of performance, probably because an acute increase in those they had in circulation from supplementation had spared those stored. These results revealed a significant improvement in the overall antioxidant reserve at the end of the recovery period, based on an increase in the defence system from three different available circulating reserves (glycosides, sulphates, and glucuronides; data not shown) associated with strengthening the main enzyme-based antioxidant defence system. The supplement probably contributed to spare oxidative homeostasis. As previously demonstrated here, the association between polyphenols from three different botanicals is possibly more efficient at reducing oxidative stress than a singular polyphenol [48,49,50]. This reflects the complex nature of the antioxidant defence system whereby each antioxidant is most efficient at quenching a certain type of free radical species and, here, synergy makes sense.

Finally, another significant marker of protection from exercise-induced stress is found in the ability of supplemented subjects to stabilize plasma levels of LDH within the healthy range, while levels for individuals in the placebo-supplemented group significantly increased by 59.2%, thereby demonstrating the deleterious effects of physical activity on red blood cells when athletes are not sufficiently protected.

## 5. Conclusions

This prospective clinical investigation on PerfLoad^®^ demonstrates that a natural and safe supplement is undoubtedly able to, similarly to training benefits, help improve metabolic and physiological pathways involved in the development of maximum metabolic power during high-intensity exercise. Indeed, supplementation is associated with a significant increase in total power output, maximal peak power output, and average power developed, without inducing either more fatigue or greater heart rate. Instead, the vascular system becomes even more well-maintained and oxidative homeostasis is stabilized. The basis for the efficiency of the supplement appears to be the synergistic activity of bioactive natural compounds, mainly polyphenols and caffeine from the three botanical extracts. Highlighted within an increasing body of research, it appears that some of these polyphenols are linked to improved endothelial function. During a pivotal clinical study to confirm our findings, further investigations will have to be conducted in attempts to elucidate the relationship between the bioavailability of polyphenol metabolites from PerfLoad^®^ supplementation and flow-mediated dilation, and especially its effects on stroke volume and the functionality of capillaries.

## Figures and Tables

**Table 1:** Bioactive content for a 1000-mg daily dose of the supplement.

Bioactive	Dose
Total polyphenols	290 mg
Bioflavonoids	170 mg
Punicalagins	100 mg
Trans-resveratrol	20 mg
Caffeine	120 mg

**Table 2:** Baseline participant characteristics.

Characteristics	All Volunteers
*N*	15
Age (years, mean ± SD)	22.2 ± 2.2
Height (cm, mean ± SD)	178.1 ± 4.3
Weight (kg, mean ± SD)	75.1 ± 6.6
BMI (kg/m^2^, mean ± SD)	22.3 ± 1.9

**Table 3:** Peak power (W) and average power (W) measured during, and fatigue index (%) and heart rate (BPM), measured just after, a 30-s WAnT bout. Values are means ± SD, *n* = 15 (placebo) or *n* = 15 (PerfLoad^®^). * Superscript indicates an intergroup difference between PerfLoad^®^ and placebo, *p* < 0.05.

Variables	*Placebo* Group	PerfLoad^®^ Group	*p* Value
Peak power (W, mean ± SD)	734 ± 104.7	761 ± 92.1 *	0.048
Average power (W, mean ± SD)	586 ± 78.9	615 ± 75.4 *	0.025
Fatigue index (%, mean ± SD)	38 ± 12.9	39 ± 10.4	0.395
Heart rate (bpm, mean ± SD)	129 ± 15.1	132 ± 25.9	0.344
